# Upregulated SLC27A2/FATP2 in differentiated thyroid carcinoma promotes tumor proliferation and migration

**DOI:** 10.1002/jcla.24148

**Published:** 2021-12-02

**Authors:** Kaixiang Feng, Runsheng Ma, Hongqiang Li, Keyu Yin, Gongbo Du, Xin Chen, Zhen Liu, Detao Yin

**Affiliations:** ^1^ Department of Thyroid Surgery The First Affiliated Hospital of Zhengzhou University Zhengzhou China; ^2^ Department of Thyroid Surgery, Key Discipline Laboratory of Clinical Medicine of Henan Zhengzhou China; ^3^ Academy of Medical Sciences Zhengzhou University Zhengzhou China; ^4^ College of Nursing Lanzhou University Lanzhou China

**Keywords:** biomarker, differentiated thyroid carcinoma, oncotherapy target, SLC27A2, tumor proliferation and invasion

## Abstract

**Background:**

Differentiated thyroid carcinoma (DTC) accounts for the vast majority of thyroid cancer (TC) cases. The rapidly increasing incidence of TC requires the urgent identification of new diagnostic and therapeutic targets. Solute carrier family 27 member 2 (SLC27A2/FATP2) plays an essential role in lipid biosynthesis and fatty acid transport. Recent studies have confirmed its involvement in a variety of diseases, including cancer.

**Methods:**

In this study, the expression of SLC27A2 was analyzed in cancer and paracancerous tissue samples from 98 thyroid cancer patients, and we performed ROC analysis to confirm the diagnostic value. CCK8, Transwell, and other methods were used to study its effect on DTC, and the mechanism of SLC27A2 was investigated by RNA sequencing and Western blot.

**Results:**

The expression of SLC27A2 was upregulated in both DTC tissues and cell lines and was correlated with clinical progression. In vitro studies further confirmed that SLC27A2 knockdown attenuated the proliferation and invasion of DTC cells. Through RNA sequence analysis and gene set enrichment analysis, we found that the MAPK pathway is the main downstream signaling pathway for the regulation by SLC27A2. SLC27A2 affects cell proliferation and differentiation by inducing changes in the proto‐oncogene C‐FOS.

**Conclusions:**

Our results show that SLC27A2 plays an important role in tumor proliferation and migration, providing a new putative target for the diagnosis and treatment of TC.

## Graphical abstract

The expression level of SLC27A2 was upregulated in differentiated thyroid carcinoma tissues compared with normal adjacent tissues, and the overexpression of SLC27A2 promoted the proliferation and migration of tumors.

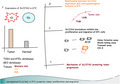

## 1 INTRODUCTION

Differentiated thyroid carcinoma (DTC) originating from the follicular epithelium represents the largest percentage (90%) of all thyroid cancers (TCs).^1^ Traditionally, DTC can be divided into papillary thyroid carcinoma (PTC) and follicular thyroid carcinoma (FTC), with most patients having a favorable prognosis after standard therapy.^2^, ^3^ However, what cannot be overlooked is that nearly one‐third of all DTCs are reported with lateral cervical lymph node metastasis (LNM).^4^ Additionally, DTC relapses in several patients, and a few patients have a 10‐year survival rate of <10%.^5^, ^6^ Recent studies regarding cancer‐associated metabolic changes, including lipid metabolism, have led to new approaches for the therapeutic intervention of TC.^7^

It is well established that tumor tissue often requires a high level of adipogenesis to support oncogenesis.^8^ For cancer cells, the specific need for lipid metabolism may be fulfilled through enhanced de novo fatty acid (FA) biosynthesis or absorption of exogenous FAs. Active transport via FA transporters (FATPs) and FA translocase (FAT/CD36) is an important pathway for the uptake of exogenous FAs by cells.^9^, ^10^ Solute carrier family 27 (SLC27) proteins are known FATPs and consist of six members, SLC27A1–SLC27A6 (FATP1‐6).^11^ FATP2 encoded by SLC27A2 is a multifunctional protein that often acts as a gatekeeper in FA transport. In the last few years, it has been found that SLC27A2 not only affects intracellular lipid homeostasis but also plays an important role in type 2 diabetes, renal fibrogenesis, and tumor progression.^12^, ^13^, ^14^ In a recently published study, Veglia et al. have demonstrated that SLC27A2 has immunosuppressive effects on neutrophils and promotes tumor growth. Results from experiments with a variety of tumor model mice suggest that SLC27A2‐specific inhibitors slow tumor growth and that the combined use of immune checkpoint inhibitors can help eliminate tumors.^14^ Targeting SLC27A2 eliminates lipid uptake and makes melanoma cells in the aging microenvironment more sensitive to targeted therapy.^15^ SLC27A2 is a beneficial therapeutic target that has shown outstanding therapeutic potential for a variety of tumors. Consequently, we decided to further elucidate its role in DTC.

In this study, the role of SLC27A2 in the development of DTC was discussed. The SLC27A2 level was significantly elevated in DTC tissues and was associated with an advanced clinical stage and lymph node metastasis. We also explored the mechanism by which SLC27A2 promotes the proliferation and invasion of DTC via the MAPK signaling pathway. We concluded that SLC27A2 plays a key role in the occurrence and development of DTC and may become a novel diagnostic and therapeutic target.

## 2 MATERIALS AND METHODS

### 2.1 Patient tissues

A total of 98 patients with DTC were randomly selected for this study between November 2019 and August 2020 at the First Affiliated Hospital of Zhengzhou University (Zhengzhou, China). All tissue samples were reviewed by endocrine pathologists and were split into two portions, with one portion preserved in liquid nitrogen and the other fixed in 10% formalin. All participants provided written informed consent. Ethical approval for this study was obtained from the Ethics Review Committee of the First Affiliated Hospital of Zhengzhou University (2020‐KY‐392).

### 2.2 Gene expression datasets

GEPIA (http://gepia2.cancer‐pku.cn/) was used to analyze the expression of SLC27A in 512 thyroid cancer tissues and 337 normal thyroid tissues from TCGA (The Cancer Genome Atlas) and GTEX (The Genotype‐Tissue Expression) databases. GSE129562 and GSE33630 data sets were downloaded from GEO (Gene Expression Omnibus Database) database, where GSE129562 contained eight tumors and eight normal tissues, and GSE33630 contained 49 tumors (PTC) and 45 normal tissues. The expression value of SLC27A2 was obtained after data processing and further analysis.

### 2.3 Quantitative real‐time PCR

Total RNA was extracted from frozen DTC tissues and cells using the TRIzol reagent (Invitrogen), and the Prime Script RT reagent kit with gDNA Eraser (Takara) was used to synthesize cDNA. Quantitative real‐time PCR was performed using the SYBR Green Detection kit (Takara). The relative mRNA expression was determined by the $2^{‐\Delta \Delta \text{CT}}$ method and β‐actin was used as the reference gene for normalization. Experiments were repeated three times. Detailed primer sequences were as follows:

SLC27A2: fwd: 5′‐CTCTTGCCTTGCGGACTAAAT‐3′, rvs: 5′‐CCTCGTAAGCCATTTCCCAGT‐3′;

β‐actin: fwd: 5′‐GTGCCAAAATGCTCAAGGAAAT‐3′, rvs: 5′‐GAAGGGCAGCTTTCTTTGTGAC‐3′.

### 2.4 Immunohistochemistry

Tumor tissues were obtained via surgical excision from patients at the First Affiliated Hospital of Zhengzhou University. First, the tissue sections were deparaffinized and rehydrated, and antigen retrieval was conducted in citrate buffer at pH 6.0 for 10 min. Next, the slides were incubated with the primary antibody. Diaminobenzidine (DAB) staining was used to detect immunoreactivity. The results were examined using Zeiss LSM 710 Confocal Microscope (Zeiss).

### 2.5 Cell culture and transfection

An immortalized human thyroid follicular epithelial cell line (Nthy‐ori 3–1), a human thyroid squamous cell carcinoma cell line (SW‐579), human PTC cell lines (TPC‐1 and B‐CPAP), and a human FTC cell line (FTC‐133) were acquired from the Shanghai Cell Biochemical Institute (Shanghai, China). They were cultured in Roswell Park Memorial Institute (RPMI)‐1640 medium supplemented with 10% fetal bovine serum (FBS) (Gemini). All the cells were incubated in a humidified incubator at 37°C with 5% CO_2_ and were tested to be free of mycoplasma contamination.

Lentivirus shRNAs were provided by Genechem (Shanghai, China). The targeting sequence of lenti‐sh‐SLC27A2 was as follows: 5′‐CCATACTTCTTCCAGGACATA‐3′. The TPC‐1 and FTC‐133 cells lines were cultured along with the lentivirus shRNAs in 6‐well culture plate at 37 °C, according to the manufacturer's instructions. After the successful transfection of the lentivirus carrying an empty vector (shNC) or sh‐SLC27A2 (shSLC27A2), the cells were cultured in the RPMI‐1640 medium containing 2 μg/ml of puromycin for one week, followed by evaluation of the transfection efficiency for the subsequent assays.

### 2.6 Western blot

Proteins from the cells were isolated with RIPA buffer (Cwbiotech) containing 1% phenylmethylsulfonyl fluoride (PMSF) (Cwbiotech). Equal amounts of protein samples were separated via 10% sodium dodecyl (lauryl) sulfate–polyacrylamide gel electrophoresis (SDS‐PAGE) and transferred to polyvinylidene fluoride (PVDF) membranes (Merck Millipore). The membranes were incubated with the indicated antibodies. Protein bands were visualized using the ChemiImager System, and ImageJ software was used to calculate the protein gray value.

### 2.7 Cell counting kit‐8 assay

The cell suspension digested in the logarithmic growth phase was evenly inoculated into 96‐well plates at a density of 1000 cells per well. The cells were incubated for 24, 48, 72, and 96 h, and 10 µl of cell counting kit‐8 (CCK‐8) solution (Dojindo) was then added to each well. After incubating the plates in an incubator for 2 h, the absorbance was measured at 450 nm using a Universal Microplate Reader.

### 2.8 Colony formation assay

Six hundred cells per well were seeded in 6‐cm Petri dishes for 2 weeks. After fixing with paraformaldehyde and staining with crystal violet, photographs were taken, and the number of colonies was calculated using the ImageJ software.

### 2.9 Wound healing assay

Sterile pipette tips were used to scratch the cell surface when the cells filled the 6‐well culture plate and were firmly attached to the wall. The cells were then washed with phosphate‐buffered saline and cultured in serum‐free RPMI‐1640 medium for 48 h. Images were captured using an inverted microscope (Olympus).

### 2.10 Transwell assay

Transwell chambers and 24‐well culture plates (Corning) were used for the transwell assay. The chambers were precoated with Matrigel matrix (BD, New Jersey, USA) for the invasion assay. Cell density was adjusted to 1 × 10^5^ cells/well in 200 µl serum‐free RPMI‐1640 medium, and the cells were seeded in the upper chamber. A total of 600 µl of the complete medium (containing 10% FBS) was inoculated in the lower chamber and incubated at 37°C for 24 h. The upper chamber was then cleaned, and the cells on the upper layer were erased. After fixing with formaldehyde and staining with 0.1% crystal violet, the cells were observed under an inverted microscope (Olympus). Finally, the ImageJ software was used to analyze and count the cells. For the transwell migration assay, the same method was used without the addition of Matrigel matrix (BD).

### 2.11 RNA sequence analysis

Three groups of TPC‐shNC and TPC‐shSLC27A2 cell samples were collected for RNA sequencing (RNA‐seq). The NEBNext^®^ rRNA Depletion Kit (New England Biolabs, Inc.) was used to remove the rRNA from total RNA of the samples. Next, RNA libraries were constructed using the NEBNext^®^ Ultra™ II Directional RNA Library Prep Kit (New England Biolabs Inc.), and their quality was controlled using the BioAnalyzer 2100 system (Agilent Technologies, Inc.). Library sequencing was performed on an Illumina HiSeq instrument, with 150‐bp paired‐end reads. RNA high‐throughput sequencing was performed by Cloud‐Seq Biotech.

### 2.12 Statistical analysis

Statistical analysis was performed using the GraphPad Prism 6 software (GraphPad Prism) and SPSS 25.0 software (IBM). Clinicopathological features of patients with DTC were analyzed via chi‐square analysis. Statistical differences between two groups were evaluated via the Student's *t* test. Data were presented as mean ± SD. *p* < 0.05 was considered statistically significant.

## 3 RESULTS

### 3.1 Expression of SLC27A2 is elevated in DTC

**FIGURE 1:**
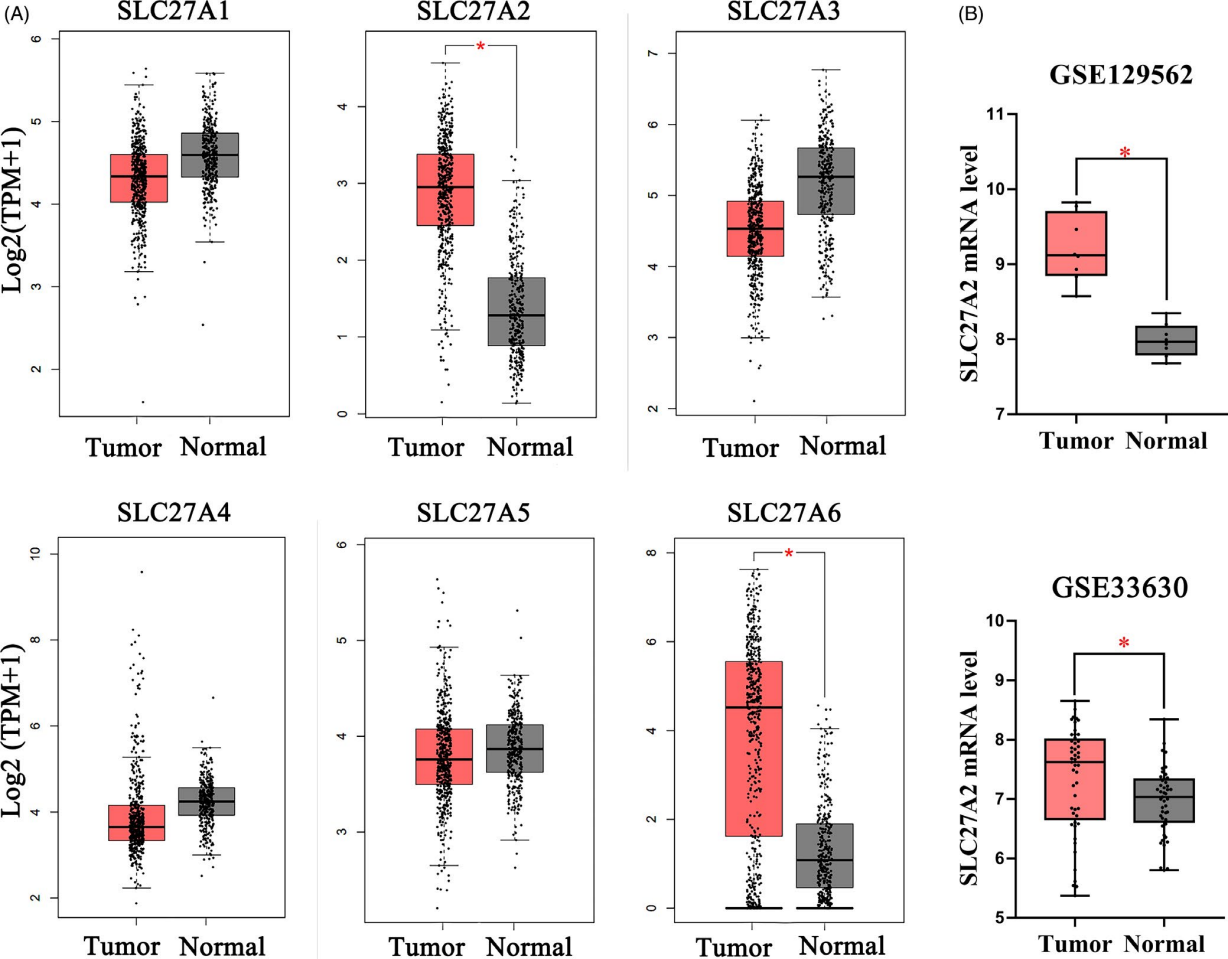
SLC27A2 is overexpressed in thyroid cancer. (A) The expression of SLC27 family members in thyroid cancer was analyzed using the GEPIA website; SLC27A2 and SLC27A6 were upregulated in thyroid cancer tissues compared with those in the adjacent normal tissues. (B) Gene Expression Omnibus datasets GSE129562 and GSE33630 showed that SLC27A2 was significantly increased in tumor tissues than that in non‐tumor tissues. *p < 0.05

**FIGURE 2:**
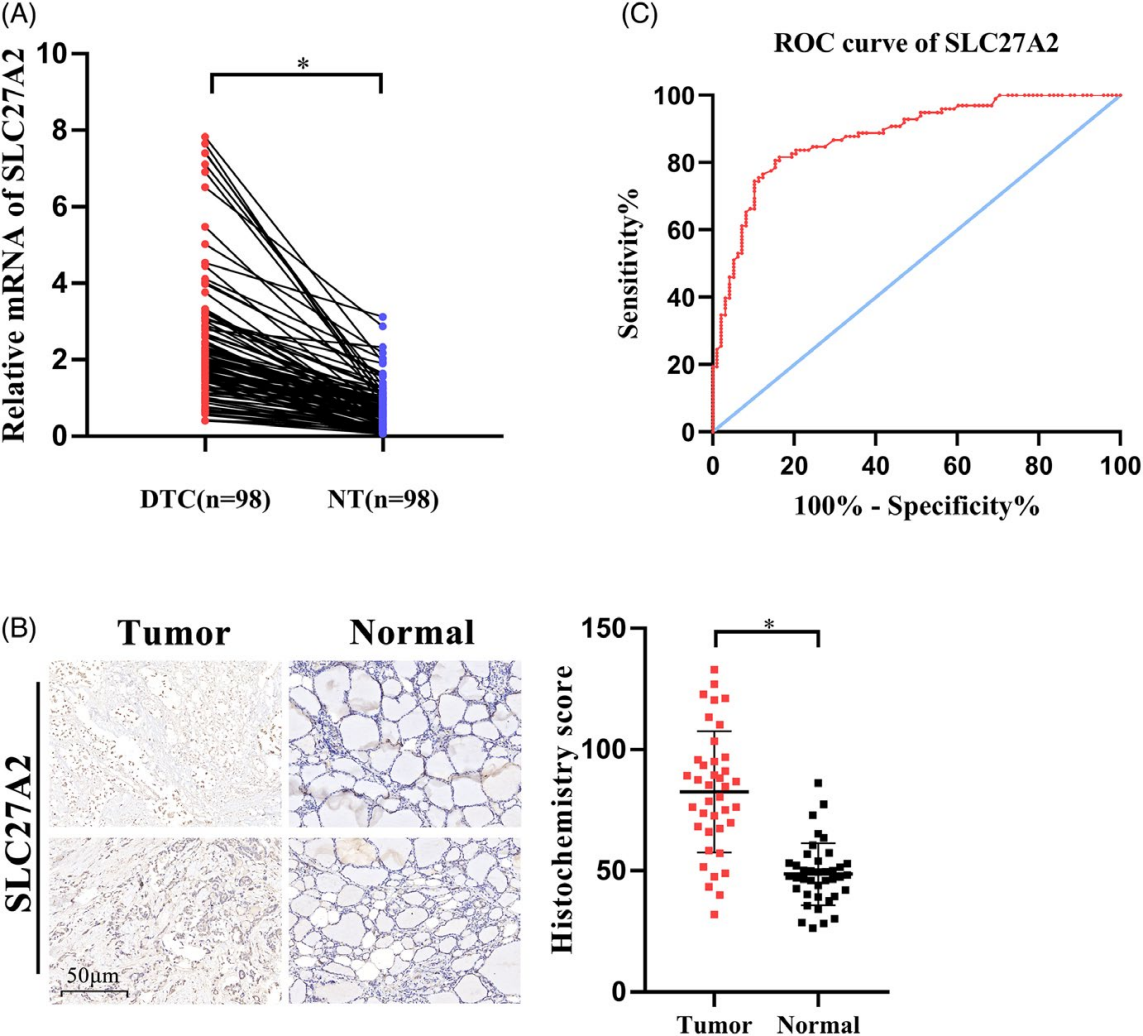
Validation of the overexpression of SLC27A2 in differentiated thyroid carcinoma (DTC). (A) The mRNA level of SLC27A2 in DTC tumor tissues was higher than that in paracancerous tissues. (B) IHC staining of SLC27A2 in DTC tissues and adjacent non‐tumor tissues. (C) SLC27A2 was a favorable diagnostic biomarker in the diagnosis of DTC, with an AUC = 0.880 (95% CI: 0.833–0.927, *p* < 0.001)

In order to identify specific proteins of the SLC27 family involved in TC, we searched TCGA and GTEx databases and found that SLC27A2 and SLC27A6 were overexpressed in TC (Figure 1A). We further analyzed GSE129562 and GSE33630 datasets (Figure 1B) and observed that SLC27A2 expression was elevated in DTC tissues compared with that in the matched non‐cancer tissues. To confirm the accuracy of the data, RT‐qPCR was used to preliminarily verify the expression of SLC27A2 in the tissues of 98 patients with DTC. Compared with that in the adjacent normal tissues, SLC27A2 was significantly upregulated in DTC tissues at the mRNA level (Figure 2A). Additionally, we examined SLC27A2 expression in DTC and para‐carcinoma samples via the Immunohistochemistry (IHC) assay (Figure 2B). The results showed that the staining strength of SLC27A2 was significantly higher in tumor tissues (*p* < 0.05) than that in the adjacent normal tissues.

### 3.2 SLC27A2 in DTC tissues was correlated with tumor stage and LNM

We assessed the relationship between the clinicopathological features of DTC and SLC27A2 expression by analyzing its mRNA levels in DTC and the adjacent normal tissues. Bound by median expression, they were divided into high and low groups. We found that SLC27A2 was not related to sex or age (*p* > 0.05) but was significantly correlated with tumor size (*p *= 0.014), TNM stages (*p* = 0.005), LNM (*p* < 0.001), and extrathyroidal extension (*p* = 0.025) (Table 1). These results confirmed that high expression of SLC27A2 was positively correlated with the clinical progression of DTC.

**TABLE 1:** Relationship between SLC27A2 mRNA expression and clinicopathological features in DTC

Characteristics	SLC27A2 expression (%)		*p* value
	High (%)	Low (%)	
Gender			
Female	35 (71.4)	38 (77.6)	0.487
Male	14 (28.6)	11 (22.4)	
Age (years)			
<55	28 (57.1)	32 (65.3)	0.407
≥55	21 (42.9)	17 (34.7)	
Histology type			
Papillary	43 (87.8)	46 (93.9)	0.294
Follicular	6 (12.2)	3 (6.1)	
Tumor size(cm)			
<2	23 (46.9)	35 (71.4)	0.014
≥2	26 (53.1)	14 (28.6)	
LNM			
Yes	35 (71.4)	17 (34.7)	<0.001
No	14 (28.6)	32 (65.3)	
TNM staging			
I	26 (53.1)	39 (79.6)	0.005
II–IV	23 (46.9)	10 (20.4)	
Multifocality			
Yes	30 (61.2)	22 (44.9)	0.105
No	19 (38.8)	27 (55.1)	
Extrathyroidal extension			
Yes	19 (38.8)	9 (18.4)	0.025
No	30 (61.2)	40 (81.6)	

Abbreviations: DTC, differentiated thyroid carcinoma; LNM, lymph node metastasis.

### 3.3 Application of SLC27A2 in the diagnosis of DTC

To evaluate the diagnostic utility of SLC27A2, we performed receiver operating characteristic (ROC) curve analysis by comparing the mRNA levels of SLC27A2 between DTC tumor tissues and adjacent normal tissues (Figure 2C). The area under the curve (AUC) was 0.880 (95% CI = 0.833–0.927, *p* < 0.001), and the sensitivity and specificity were 80.6% and 84.7%, respectively (Youden index = 0.653). These results suggest that SLC27A2 may be an effective biomarker for the diagnosis of DTC.

### 3.4 SLC27A2 knockdown inhibits the proliferation and migration of DTC cells

**FIGURE 3:**
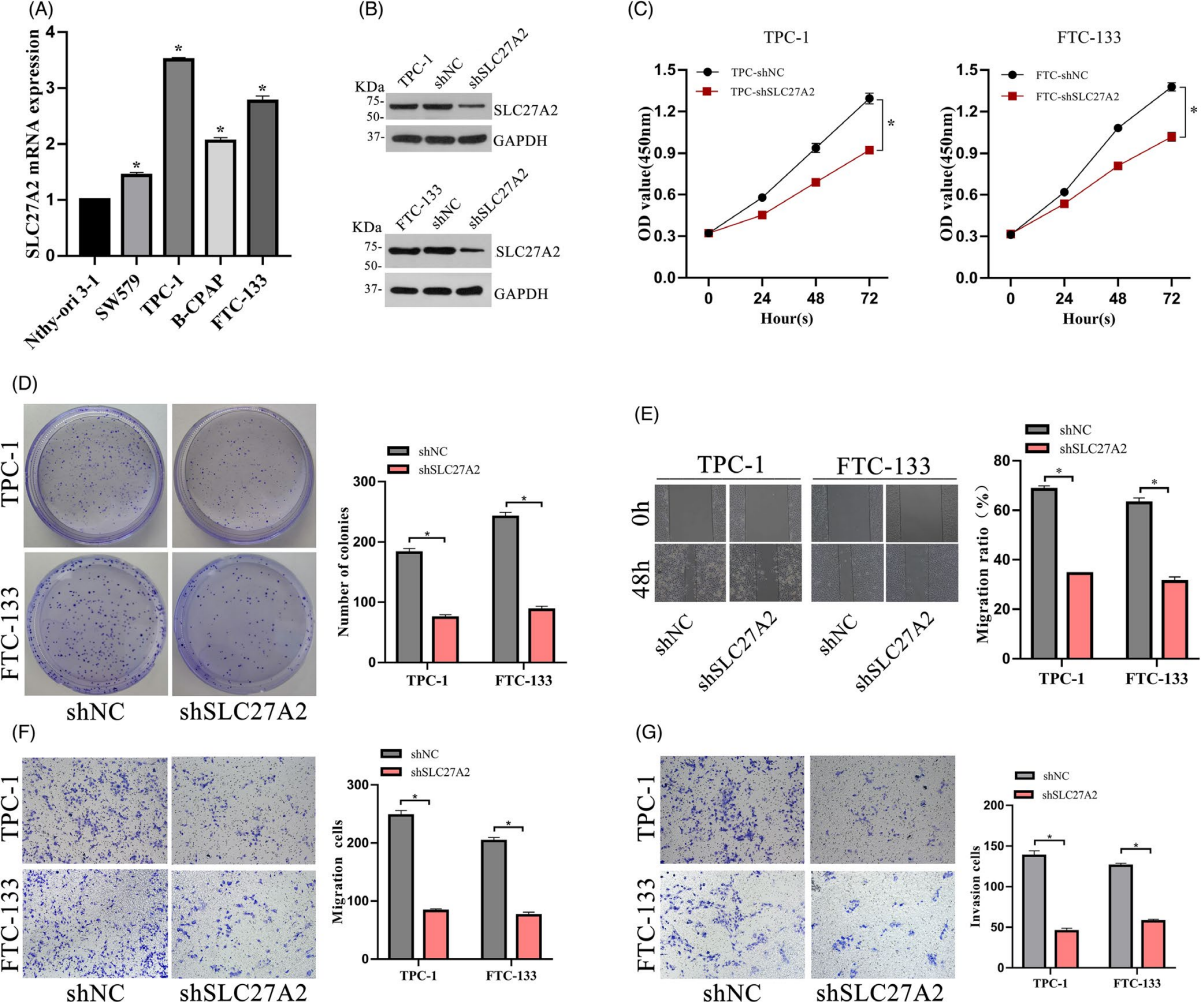
SLC27A2 silencing inhibited the proliferation and migration of DTC. (A) Elevated levels of SLC27A2 were found in various thyroid cancer cell lines compared with those in the Nthy‐ori 3–1 cell line. (B) Western blot analysis showed that SLC27A2 protein was downregulated by approximately 60% after transfection, *p* < 0.05. (C‐D) The proliferation ability of TPC‐1 and FTC‐133 cells in the SLC27A2‐silenced group was decreased compared with that in the control group, as determined via the CCK‐8 and colony formation assays. (E‐F) Wound healing assay and transwell assay showed that cell migration was decreased in the SLC27A2‐silenced group compared with that in the control group. (G) Compared with that of the control group, the cell invasion ability of the SLC27A2‐silenced group decreased significantly. *p < 0.05

To explore the effect of SLC27A2 on tumor growth, the expression levels of SLC27A2 in a collection of thyroid cell lines were detected via RT‐qPCR (Figure 3A). We transfected SLC27A2‐shRNA lentiviral vectors into the TPC‐1 and FTC‐133 cell lines, which had high SLC27A2 expression, and shNC was used as the control. The efficiency of knockdown was confirmed via Western blot (Figure 3B). Knockdown of SLC27A2 in TPC‐1 and FTC‐133 cells suppressed cell proliferation, as shown by the CCK‐8 assay (Figure 3C). Furthermore, the colony formation rate was significantly decreased in SLC27A2 knockdown group (Figure 3D). Next, to determine the role of SLC27A2 in DTC migration, wound healing and transwell assays were performed (Figure 3E‐F), and their results showed that SLC27A2 knockdown significantly reduced the migration of DTC cells compared to that in the negative control group. In addition, transwell invasion assays showed that the invasion ability of DTC cells was also weakened after SLC27A2 knockdown (Figure 3G).

### 3.5 SLC27A2 knockdown inhibited the MAPK pathway and reduced tumor progression

**FIGURE 4:**
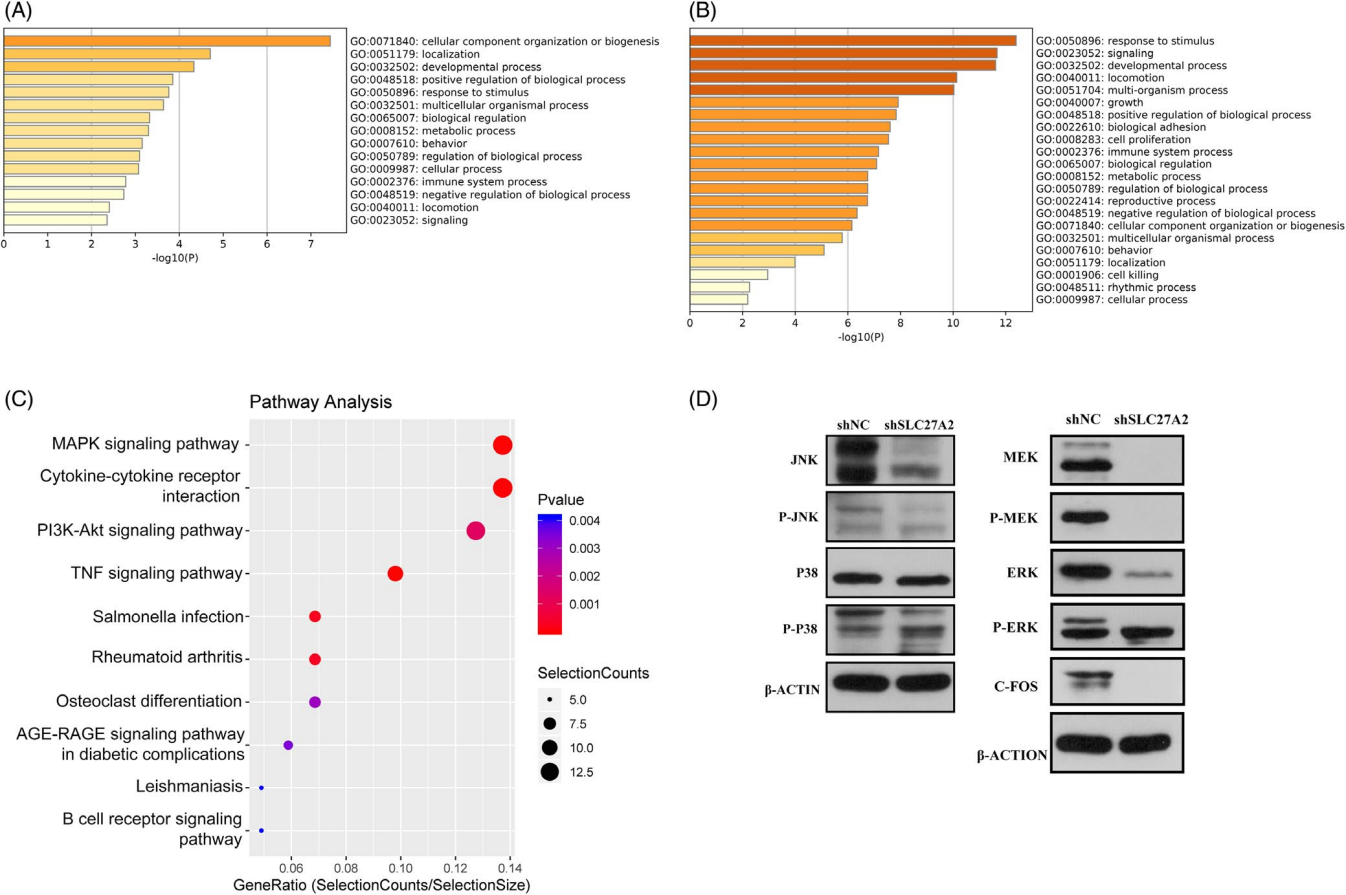
Silencing SLC27A2 in DTC cells affects the MAPK pathway and weakens tumor development. (A) GO function analysis of upregulated differential genes was performed in *Metascape*. (B) GO function was analyzed for the downregulated differential genes. (C) KEGG pathway analysis, downregulated genes after SLC27 knockdown were significantly enriched in MAPK signaling pathway, PI3K‐Akt signaling pathway, and other pathways. (D) The protein expression of the MAPK pathway key kinases in TPC‐1 cells was detected. * *p* < 0.05

Experimental and control cell samples were selected for RNA‐seq. Differentially expressed genes (DEGs) between the two groups were further filtered out (|log2Fold change| ≥1 and *p*≤0.05) and found 125 significantly upregulated and 223 significantly downregulated DEGs (Table 2). The Gene Ontology (GO) analysis of these genes was visualized by *Metascape*, and it was found that the downregulated DEGs were mainly involved in biological processes such as "development process," "locomotion," "cell proliferation," "biological adhesion," "growth" and other biological processes (Figure 4A‐B).^16^ This is consistent with our previous results of the proliferation and migration experiments. Kyoto Encyclopedia of Genes and Genomes (KEGG) analysis of DEGs showed that after SLC27A2 gene silencing, the downregulated DEGs were mainly enriched in the MAPK pathway (Figure 4C).

**TABLE 2:** Significantly differentially expressed genes after downregulation of SLC27A2 (TOP10)

Gene	Log2 (fold change)	*p* value	Regulation
KLRC3	−6.78146	0.0361	Down
FOSB	−6.40491	0.00005	Down
FPGT‐TNNI3K	−6.1385	0.0046	Down
EGR1	−5.61296	0.00005	Down
TSACC	−5.24402	0.01085	Down
NTRK1	−4.5717	0.00095	Down
FGFR2	−4.53243	0.0367	Down
FOS/C‐ FOS	−4.32977	0.00005	Down
DDR2	−4.31706	0.0002	Down
NKG2‐E	4.78722	0.02865	Up
PLXDC1	4.62632	0.00005	Up
CNGB1	3.92734	0.00005	Up
SLC25A21	3.81088	0.0093	Up
KCNIP2	3.33899	0.00005	Up
TCF4	2.96333	0.00005	Up
LPHN2	2.57534	0.00005	Up
RP11‐47I22.3	2.45002	0.0384	Up
SMIM10	2.28377	0.0253	Up
PPFIA4	2.22731	0.00005	Up

Next, we identified the major kinases in the MAPK pathway in TPC‐1 by Western blot and found that JNK and ERK protein levels were significantly inhibited after downregulation of SLC27A2 in TPC‐1, followed by analysis of the classical MAPK pathway, where ERK was located. Further experiments showed that SLC27A2 mainly caused a change in C‐FOS expression through the classical MAPK pathway (Figure 4D).

## 4 DISCUSSION

Dysregulation of SLC27 family proteins often leads to abnormal FA transport and lipid accumulation. Over the past few years, it has been found that their members are widely involved in the progression of cancer. For instance, SLC27A1 has recently been reported to be highly expressed in breast cancer and may contribute toward an innovative therapeutic strategy.^17^ Likewise, SLC27A4 has been found to be associated with the progression of clear cell renal cell carcinoma and breast cancer,^18^, ^19^ SLC27A5 deficiency causes a series of reactions in hepatoma cells, such as increase in polyunsaturated lipids and lipid peroxidation, leading to the proliferation of tumor cells. It provides a potential prognostic marker and treatment options for HCC.^9^, ^20^ TC is a multi‐factorial disease, and abnormal lipid metabolism has been reported in TC tissues as well. Excessive accumulation of FAs provides a constant source of energy for the growth of tumor cells.^7^, ^21^ In recent years, the incidence of DTC has increased rapidly; however, its therapeutic efficiency of its treatment still needs to be improved. Some patients with DTC remain difficult to treat and have a high risk of death, especially those exhibiting iodine treatment resistance, distant metastasis, and tumor recurrence.^22^, ^23^, ^24^ This study on SLC27A2 may be an important milestone in establishing the association between FAs and DTC.

The search through TCGA and other databases showed that SLC27A2 and SLC27A6 were significantly overexpressed in TC. Likewise, another study supports the differential expression of SLC27A6 in PTC.^25^ Based on the intensive exploration and extensive therapeutic potential of SLC27A2 in other tumors, this study focused on the analysis of SLC27A2 in DTC, intending to lay a foundation for future research. Our study highlights the role of SLC27A2 in promoting tumor proliferation and invasion. Through a series of experimental investigations, we showed that SLC27A2 expression is upregulated in DTC tissues and cells, and its high expression is related to TNM staging, LNM, and other clinical characteristics of DTC. SLC27A2 promoted the proliferation and migration of DTC cells in vitro. This was also confirmed by a subsequent RNA‐seq analysis. Additionally, we evaluated the diagnostic efficacy of SLC27A2, suggesting that it may be an effective diagnostic marker for DTC, although there was no significant difference in SLC27A2 expression between PTC and FTC tissues (*p* = 0.294).

The abnormal expression of SLC27A2 in DTC and its role in tumor progression encouraged us to study it further. The important roles of the MAPK and PI3K‐Akt pathways in DTC tumorigenesis have been confirmed.^26^, ^27^ RNA‐seq data revealed that SLC27A2 gene knockdown had an inhibitory effect on these pathways, and we focused on the most obvious MAPK pathway. The extensive changes in secondary molecules synergize and amplify the MAPK pathway, thus increasing the oncogenic activity of TC.^26^ C‐FOS is an important proto‐oncogene that regulates cell proliferation, differentiation, and metastasis. C‐FOS also plays an important role in the progression of TC. Studies have found that C‐FOS is highly expressed in PTC and is associated with LNM.^28^ Since C‐FOS was one of the differentially expressed genes according to sequencing data, a possible mechanism of SLC27A2 may comprise continuous amplification of the signal through MAPK pathway, causing a change in the C‐FOS expression levels and the eventual proliferation and migration of DTC.

It is worth noting that this study has several limitations: We did not discuss the Hurthle cell tumor owing to its relative rarity and account for a small proportion of DTC. We discussed the role of SLC27A2 in DTC rather than in PTC and FTC individually, both of which are sources of follicles. The application of SLC27A2 needs further study in future, such as the evaluation of the efficacy of SLC27A2/FATP2 inhibitor in the treatment of advanced TC.

In conclusion, this study revealed the importance of SLC27A2 in the occurrence and development of DTC. SLC27A2 may promote tumor proliferation and migration through the change of C‐FOS expression, and our study provides a potential prognostic indicator and a treatment method for patients with DTC.

## CONFLICTS OF INTEREST

The authors declare that they have no competing interests.

## AUTHOR CONTRIBUTIONS

DTY proposed research ideas and provided guidance. RSM and HQL designed the experimental scheme. KXF, KYY, and GBD carried out the experimental implementation and data analysis. KXF and RSM wrote the manuscript together. XC and ZL critically revised the manuscript. All authors read and approve the final manuscript.

## Data Availability

The data that support the findings of this study are available from the corresponding author upon reasonable request.
